# A longitudinal examination of the measurement properties and invariance of the Sleep Condition Indicator in Chinese healthcare students

**DOI:** 10.1186/s12888-024-05844-7

**Published:** 2024-07-22

**Authors:** Runtang Meng, Yiwei Ying, Yi Luo, Mengyi Huang, Christopher B. Miller, Yuhuan Xie, Yuxin Jia, Lianxia Fan, Wukang Chen, Jiayu Yi, Nongnong Yang, Jiale Xu, Chen Jiang, Liping Lu, Haiyan Ma, Karen Spruyt, Esther Yuet Ying Lau

**Affiliations:** 1 School of Public Health, Hangzhou Normal University, Hangzhou 311121, Zhejiang China; 2 Engineering Research Center of Mobile Health Management System, Ministry of Education, Hangzhou, Zhejiang China; 3 School of Nursing, Hangzhou Normal University, Hangzhou, Zhejiang China; 4 School of Nursing, Ningbo College of Health Sciences, Ningbo, Zhejiang China; 5 Big Health Ltd., London, UK; 6 School of Clinical Medicine, Hangzhou Normal University, Hangzhou, Zhejiang China; 7 Université Paris Cité, NeuroDiderot, INSERM, Paris, France; 8 Sleep Laboratory, Department of Psychology, The Education University of Hong Kong, Hong Kong, China; 9 Centre for Psychosocial Health, The Education University of Hong Kong, Hong Kong, China; 10 Centre for Religious and Spirituality Education, The Education University of Hong Kong, Hong Kong, China

**Keywords:** Sleep Condition Indicator, Insomnia symptoms, Measurement properties, Measurement invariance, Observational longitudinal design, Healthcare students

## Abstract

**Background:**

The Sleep Condition Indicator (SCI), an insomnia measurement tool based on the updated Diagnostic and Statistical Manual of Mental Disorders, Fifth Edition (DSM-5) criteria with sound psychometric properties when applied in various populations, was evaluated here among healthcare students longitudinally, to demonstrate its measurement properties and invariance in this particularly high-risk population.

**Methods:**

Healthcare students of a Chinese university were recruited into this two-wave longitudinal study, completing the simplified Chinese version of the SCI (SCI-SC), Chinese Regularity, Satisfaction, Alertness, Timing, Efficiency, Duration (RU_SATED-C) scale, Chinese Patient Health Questionnaire-4 (PHQ-4-C), and sociodemographic variables questionnaire (Q-SV) between September and November 2022. Structural validity, measurement invariance (MI), convergent and discriminant validity, internal consistency, and test–retest reliability of the SCI-SC were examined. Subgroups of gender, age, home location, part-time job, physical exercise, and stress-coping strategy were surveyed twice to test cross-sectional and longitudinal MI.

**Results:**

We identified 343 valid responses (62.9% female, mean age = 19.650 ± 1.414 years) with a time interval of seven days. The two-factor structure was considered satisfactory (comparative fit index = 0.953–0.989, Tucker–Lewis index = 0.931–0.984, root means square error of approximation = 0.040–0.092, standardized root mean square residual = 0.039–0.054), which mostly endorsed strict invariance except for part-time job subgroups, hence establishing longitudinal invariance. The SCI-SC presented acceptable convergent validity with the RU_SATED-C scale (*r* ≥ 0.500), discriminant validity with the PHQ-4-C (0.300 ≤ *r* < 0.500), internal consistency (Cronbach’s alpha = 0.811–0.835, McDonald’s omega = 0.805–0.832), and test–retest reliability (intraclass correlation coefficient = 0.829).

**Conclusion:**

The SCI-SC is an appropriate screening instrument available for assessing insomnia symptoms among healthcare students, and the promising measurement properties provide additional evidence about validity and reliability for detecting insomnia in healthcare students.

**Supplementary Information:**

The online version contains supplementary material available at 10.1186/s12888-024-05844-7.

## Background

Insomnia disorder is defined as a persistent difficulty with sleep initiation, duration, or consolidation that occurs despite adequate opportunities and circumstances for sleep and results in concern, dissatisfaction, or perceived daytime impairment, such as fatigue, decreased mood or irritability, general malaise, or cognitive impairment [1]. To date, insomnia is globally considered one of the most prevalent sleep disorders and costly public health problems [2]. As estimated, up to 10% of the general population met the diagnostic criteria for insomnia, while up to 30% experienced some symptoms of insomnia [3]. Given its pervasiveness, the detrimental effects of insomnia have been increasingly documented. Several studies identified insomnia as a significant risk factor for depression and anxiety and even documented association with suicidality [4–6]. A meta-analysis showed that insomniacs have a severely impaired quality of life, greatly affecting their ability to function well at work, in their health, and in their social lives [7]. However, independently assessing insomnia is rarely straightforward, often involving multiple interacting psychiatric and medical comorbidities [8]. Given its high prevalence and harmful effects, an effective diagnosis of insomnia should be considered a key step in promoting optimal sleep health.

Insomnia can only be assessed via subjective measures, and objective measures may be useful for uncovering further sleep disorders. Historically, three primary sources of information have been utilized in diagnosing insomnia: (i) *sleep history*, including quantitative and qualitative sleep information, and perspectives of patients about waking function; (ii) *typical two-week sleep diary*, as a collection of nightly sleep estimates; (iii) *polysomnography*, which provides objective sleep assessments and may detect the presence of further sleep disorders [9]. Epidemiologic studies have indicated that in population where dissatisfaction with sleep is prevalent, clinical diagnoses of sleep disorders are often synchronized [10]. Thus, subjective patient reports offer precise diagnostic information given symptom-based definitions [9]. In addition, objective measuring is a time-consuming and costly process [11]. To work around this, there is a greater need for a reliable, valid, and brief screening instrument to provide further empirical support for insomnia evaluation.

A plethora of subjective measurements exist for the identification of insomnia [12–16], some examples are the Pittsburgh Sleep Quality Index (PSQI) and Insomnia Severity Index (ISI), both of which are of different natures and purposes. Nevertheless, the PSQI assesses general sleep quality in the general population [17], while the ISI measures insomnia symptoms not according to the Diagnostic and Statistical Manual of Mental Disorders, Fifth Edition (DSM-5), hence without including the aspect of duration [18]. There is currently a lack of scale developed specifically on the updated DSM-5 criteria. The Sleep Condition Indicator (SCI) is a versatile, user-friendly, and brief instrument developed on the updated DSM-5 criteria [19]. It is designed for subjective self-reporting of insomnia and monitors a dimensional perspective on sleep quality, a visual profile of night-time and daytime symptoms, and indicative cut-off points for clinically significant insomnia [19].

Since the release of the English version in 2012, the SCI has been adapted and validated in over 12 languages, including Romanian (2013) [20], English (2014) [19], Italian (2015) [21], traditional Chinese (2017) [11], French (2017) [22], Korean (2018) [23], Swedish (2019) [24], Persian (2019) [25], Arabic (2021) [26], simplified Chinese (2022) [27], Indonesian (2023) [28], and Turkish (2024) [29]. However, there has yet to be validation of longitudinal measurement invariance (LMI) in all existing versions, nor has there been any evaluation in healthcare students. A previous study reported some key measurement properties of the simplified Chinese version of the SCI (SCI-SC) through COnsensus-based Standards for the selection of health Measurement INstruments (COSMIN), containing promising structural validity, construct (convergent and divergent) validity, internal consistency, and the most key―cross-sectional measurement invariance (MI) [27]. Nonetheless, longitudinal evidence for the SCI-SC has not been provided, and establishing construct validity with the Chinese Sleep Quality Questionnaire (SQQ-C) solo is insufficient [11].

Healthcare students exhibit particularly serious insomnia symptoms [30], including daytime somnolence, sleep deprivation, and poor sleep quality compared to non-medical students and the general population [27, 31–33]. Alarmingly, the prevalence of insomnia continues to increase among Chinese healthcare students [34], resulting in reduced learning capacity, declarative and procedural learning, and neurocognitive functioning [35]. Excessive academic stress often worsens daytime impact on healthcare students, resulting in absence, tardiness, falling asleep during class and interference with academic achievement [36]. While these symptoms appear to be consistent with the Sleep Pattern Subscale (SPS) and Daytime Impact Subscale (DIS) of the SCI, it would be important to look into the psychometric properties empirically because Swedish undergraduate students have reported contradictory findings of unidimensionality in structural validity [24].

Three overarching goals of this study are to: (i) fill the gap in the validation of measurement properties of the SCI in healthcare students; (ii) provide psychometrics evidence of the SCI-SC, including structural validity, cross-sectional and longitudinal MI, internal consistency and test–retest reliability; (iii) explore criterion validity with the correlation between the SCI-SC and two external variables.

## Methods

### Sample and procedure

Participant recruitment took place from September to November 2022 at a university in Hangzhou, China. The study included conveniently sampled freshman, junior, and senior undergraduate students majoring in clinical medicine and preventive medicine. We used the rule of thumb of 20 subjects per item [37], along with a minimum of 300 respondents to conduct the factor analysis [38]. The inclusion criteria were healthcare students who were capable of reading simplified Chinese and communicating in Mandarin. Exclusion criteria were those who: (i) had difficulty with study process; (ii) were on long-term medical internship leave or suspension; (iii) were taking medication for sleep disorders, had psychiatric diagnoses, or had substance abuse.

This study was performed under an interval of approximately seven days, due to recommended longitudinal use of 2 to 14 days [39–41]; the below three measures and sociodemographic variables questionnaire (Q-SV) were administered at baseline (Time 1, T1) and follow-up (Time 2, T2) assessment [19, 42–44]. Well-trained investigators maintained quality control of the data collection process. Respondents filled out self-administered paper-and-pencil surveys, with their student IDs for matching questionnaires at two assessments.

This study followed the ethical standards in accordance with the Declaration of Helsinki [45].

### Measures

#### Sociodemographic variables questionnaire

The Q-SV gathered sociodemographic characteristics including gender (male and female), age, major (clinical medicine and preventive medicine), home location (urban, rural, and suburban), part-time job (yes and no), physical exercise (yes and no), stress-coping strategy [the most customary way of coping when faced with great stress (emotion-focused, solution-focused, and avoidance coping) [46]].

#### Sleep Condition Indicator (Chinese version)

The SCI is a five-point Likert scale (0–4), comprising two subscales of five items probing Sleep Pattern (SP) and three items exploring Daytime Impact (DI) [19]. Scores lie in the point range from 0 to 32, with a higher score reflecting better sleep and a lower likelihood of insomnia. An SCI score of 16 or lower may define insomnia disorder and indicate four main DSM-5 criteria are met: difficulty initiating or maintaining sleep, significant distress, frequency of sleep disturbances, and duration of sleep disturbances [26]. The SCI-SC is a reliable measure with promising psychometric properties in community residents [27].

#### Regularity, Satisfaction, Alertness, Timing, Efficiency, Duration scale (Chinese version)

The Regularity, Satisfaction, Alertness, Timing, Efficiency, Duration (RU_SATED) scale, a six-item/dimension scale consisting of sleep Regularity, Satisfaction with sleep, Alertness during waking hours, Timing of sleep, sleep Efficiency, and sleep Duration, is a generic instrument measuring sleep health and emphasizing positive effects of sleep on overall health [43, 47]. Respondents expressed their level of agreement or disagreement with each item using a five-point Likert scale (0 = never, 1 = rarely, 2 = sometimes, 3 = usually, 4 = always), summed up ranging from 0 to 24, where a higher score indicates better sleep health. Previous results supported satisfactory measurement properties of the Chinese RU_SATED (RU_SATED-C) scale [48].

#### Patient Health Questionnaire-4 (Chinese version)

The Patient Health Questionnaire (PHQ), a self-report version of the Primary Care Evaluation of Mental Disorders (PRIME-MD), was developed to assist primary care clinicians in making efficient psychiatric diagnoses [49]. As a form with fewer items, the Patient Health Questionnaire-4 (PHQ-4) consists of the Patient Health Questionnaire-2 (PHQ-2) about the diagnosis of depressive disorders and the Generalized Anxiety Disorder scale-2 (GAD-2) about two core criteria for anxiety symptoms [44]. Scored on a four-point Likert scale (0 = not at all, 1 = several days, 2 = more than half the days, 3 = nearly every day), composite scores range from 0 to 12, with higher scores indicating worse anxiety and depression symptoms [44]. Assessed directly from the PHQ Screeners website [50], the Chinese Patient Health Questionnaire-4 (PHQ-4-C) had shown adequate measurement properties in Chinese adults [51].

### Statistical approach

We applied EpiData (version 3.1) to build the database. Statistical analyses were conducted using R software (version 4.1.3) with several packages (“*lavvan* (0.6–11)” [52], “*MBESS* (4.9.1)” [53], “*irr* (0.84.1)” [54], “*semTools* (0.5–6)” [55]) and JASP software (version 0.16.1). Measurement properties were assessed compliant with COSMIN methodology [56]. The missing values were imputed with the mean (continuous variables) or median (categorical variables) when produced < 1% of total missing values [57].

#### Structural validity

The primary role of exploratory factor analysis (EFA) is to explore and identify factors that define the construct, while confirmatory factor analysis (CFA) follows EFA and theoretical knowledge to examine factor structure further [58]. In previous studies, both traditional and simplified Chinese versions showed stable and identical solutions with two dimensions (SP and DI) [11, 27], supporting in conducting CFA rather than EFA.

CFA was applied on the one-factor (obtained in the Swedish version [24]), two-factor (suggested in the traditional and simplified Chinese versions [11, 27]), and second-order factor models. We used the Maximum Likelihood Robust Estimator (MLR) method that is appropriate for ordinal data to separately determine which of the three proposed models could achieve the best goodness of fit [59]. The recommended two-indicator strategy was used to select indices [60]: (i) the chi-square (*χ*^*2*^) and *P* value; since to be sensitive in a large sample, they only were treated as secondary indicators; (ii) comparative fit index (CFI), Tucker–Lewis index (TLI), root means square error of approximation (RMSEA), and standardized root mean square residual (SRMR); threshold values of CFI and TLI ≥ 0.900, RMSEA ≤ 0.100, SRMR ≤ 0.080 were indicative of model employment [61–63].

#### Measurement invariance

MI requires that the structure of scale should not depend on measurement groups, equally reflecting constructs of interest under different groups of individuals [64]. Compared the SCI scores across subgroups based on sociodemographic variables that are closely associated with insomnia in general population (i.e., gender, age, and home location [65–67]) or in healthcare students (i.e., part-time job, physical exercise, and stress-coping strategy [68–77]). LMI was examined using longitudinal CFA (LCFA) across two occasions.

Cumulatively adding constraints, we tested configural, metric, scalar, and strict invariance, which correspondingly require the same factor structure, factor loadings, item intercepts, and item residual between subgroups [78]. The unstandardized regression coefficients, and regression coefficients and means of latent structures are comparable when metric and scalar invariance are confirmed, respectively [79]. Relied on the conventional recognized standards, changes in CFI (ΔCFI) ≤ 0.010, changes in TLI (ΔTLI) ≤ 0.010, changes in RMSEA (ΔRMSEA) ≤ 0.015, and changes in SRMR (ΔSRMR) ≤ 0.030 were considered acceptable model [80]. We have also considered changes in chi-square (Δ*χ*^*2*^), but only as a secondary indicator [81]. For all models, if at least two out of three fit indices comply with the cut-off criteria, it indicates no significant degradation in model, suggesting that MI is held [82].

#### Convergent and discriminant validity

Evidence of convergent and discriminant validity based on Spearman correlation coefficient was provided by correlating the SCI-SC scores with RU_SATED-C scale and PHQ-4-C scores, respectively. Following the COSMIN guideline, at least 75% of the hypothesis need to be within range [83]. We hypothesized:

(i) The SCI-SC scores would have a strong correlation (*|r|*≥ 0.500) with the RU_SATED-C scale scores, given that both instruments measure related constructs concerning sleep [58, 84].
(ii) The SCI-SC scores would have a moderate correlation (0.300 ≤ |*r*| < 0.500) with the PHQ-4-C scores, given that two instruments measure theoretically different constructs [58, 84].

#### Internal consistency and test–retest reliability

Internal consistency means the degree of interrelatedness between items [84]. We assessed internal consistency of the SCI-SC scores and two sub-scores using Cronbach’s alpha and McDonald’s omega [81]. Cronbach’s alpha and McDonald’s omega values > 0.700 indicated adequacy [85].

To test stability of the SCI-SC for the same respondents in the same test at different times [58], we studied test–retest reliability using intraclass correlation coefficient (ICC); in doing so, generally, ICC > 0.700 was sufficient [86]. Standard error of measurement (SEM) was evaluated as a supplement to measurement precision [86].

## Results

### Sample description

A total of 343 healthcare students were analyzed, with a total missing data rate of 0.066% and a dropout rate of less than 5%; 62.974% were female, and the mean age was 19.650 ± 1.414 (mean ± standard deviation) years. The average time interval between two occasions was 7 days + 2 h. Except for item 2, scores of seven items on the SCI-SC contented a multivariate normal distribution by checking skewness and kurtosis [87]. Additional sociodemographic characteristics can be found in Table 1, and the distribution of scores is presented in Supplementary 1: Table S1.

**Table 1:** Sociodemographic variables (*N* = 343)

Variables	*N* (%)	SCI-SC Mean (SD)	
		Baseline	Follow-up
Gender			
Male	127 (37.026)	24.520 (4.866)	24.890 (4.421)
Female	216 (62.974)	25.019 (4.701)	25.148 (4.947)
Age			
< 20 years	150 (43.732)	25.633 (4.311)	25.720 (4.466)
≥ 20 years	193 (56.268)	24.212 (5.008)	24.534 (4.915)
Major			
Clinical medicine	154 (44.898)	53.039 (17.232)	54.883 (19.518)
Preventive medicine	189 (55.102)	60.423 (17.234)	60.614 (17.246)
Home location			
Urban	156 (45.481)	24.808 (4.797)	25.026 (4.726)
Rural	101 (29.446)	24.762 (4.718)	25.119 (4.680)
Suburban	86 (25.073)	24.965 (4.803)	25.023 (4.942)
Part-time job			
Yes	33 (9.621)	23.515 (4.570)	24.485 (5.136)
No	310 (90.379)	24.974 (4.768)	25.113 (4.716)
Physical exercise			
Yes	257 (74.927)	25.019 (4.687)	25.105 (4.875)
No	86 (25.073)	24.279 (4.965)	24.895 (4.395)
Stress-coping strategy			
Emotion-focused	189 (55.102)	24.857 (4.292)	25.307 (4.137)
Solution-focused	121 (35.277)	25.306 (5.089)	25.207 (5.252)
Avoidance coping	33 (9.621)	22.970 (5.709)	23.030 (5.731)

Abbreviations: *SCI* Sleep Condition Indicator, *SC* simplified Chinese, *SD* standard deviation, *Mean (SD)* mean scores and SDs

### Structural validity

The fit indices indicated a better fit of both baseline and follow-up data to the two-factor structure (SP and DI) (CFI and TLI ≥ 0.900, RMSEA ≤ 0.100, SRMR ≤ 0.080), as presented in Table 2. Although RMSEAs approaching the critical value in the follow-up sample were cautionary, they were still within a reasonable range.

**Table 2:** CFA fit indices for three alternative models of the SCI-SC (*N* = 343)

Model	*χ*^*2*^* (df)*	*P*	CFI	TLI	RMSEA (90% CI)	SRMR
Baseline						
One-factor	192.989 (20)	< 0.001	0.825	0.755	0.156 (0.133, 0.180)	0.095
Two-factor	34.282 (19)	0.017	0.989	0.984	0.040 (0.000, 0.071)	0.039
Second-order factor	43.975 (20)	0.002	0.980	0.972	0.053 (0.021, 0.081)	0.068
Follow-up						
One-factor	251.394 (20)	< 0.001	0.807	0.730	0.181 (0.157, 0.205)	0.106
Two-factor	79.337 (19)	< 0.001	0.953	0.931	0.092 (0.066, 0.118)	0.054
Second-order factor	93.785 (20)	< 0.001	0.941	0.918	0.100 (0.075, 0.125)	0.090
Threshold	N/A	> 0.050	≥ 0.900	≥ 0.900	≤ 0.100	≤ 0.080

Abbreviations: *CFA* confirmatory factor analysis, *SCI* Sleep Condition Indicator, *SC* simplified Chinese, *χ*^*2*^ chi-square, *df* degrees of freedom, *CFI* comparative fit index, *TLI* Tucker–Lewis index, *RMSEA* root mean square error of approximation, *CI* confidence interval, *SRMR* standardized root mean square residual, *N/A*, not applicable

### Measurement invariance

#### Cross-sectional measurement invariance

An evaluation of cross-sectional measurement invariance on two-factor model, the best-fitting model within three alternative SCI-SC models, was tested. Results showed evidence for well-fixed configural, metric, scalar, and strict invariance across gender, age, home location, physical exercise, and stress-coping strategy (CFI and TLI ≥ 0.900, RMSEA ≤ 0.100, SRMR ≤ 0.080; ΔCFI and ΔTLI ≤ 0.010, ΔRMSEA ≤ 0.015, ΔSRMR ≤ 0.030) (Table 3). Although cut-off values exceeded, scalar invariance in age subgroups was still supported due to negligible changes in fit indices for strict invariance. Compared with the baseline assessment, RMSEAs in the follow-up assessment were increased to different degrees and partly fell outside the recommended range, but we still accepted such results due to at least two out of three fit indices comply with the cut-off criteria [82]. However, significant changes in CFI and TLI values were observed across part-time job subgroups for strict invariance (baseline: ΔCFI = -0.015, ΔTLI = -0.011; follow-up: ΔCFI = -0.039, ΔTLI = -0.030), the highest supporting scalar invariance. Complete MI results are shown in Supplementary 1: Table S2 and Supplementary 2 reports a more detailed description.

**Table 3:**
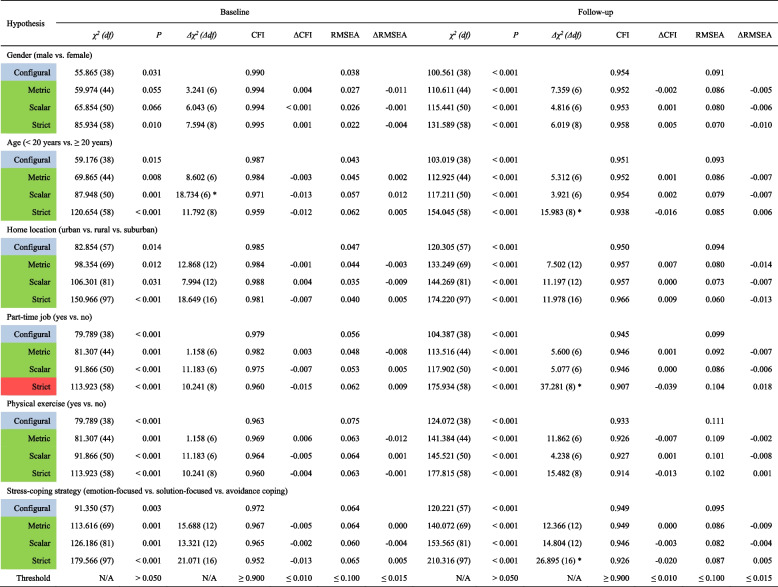
Cross-sectional measurement invariance of the SCI-SC (*N* = 343)

Notes: Table shadings of the first column represent different levels of model held: 1) Blue represents that this is the configural model; 2) Green represents that this model is fully supported; 3) Red represents that this model is unsupported; *, *P* < 0.050

Abbreviations: *SCI* Sleep Condition Indicator, *SC* simplified Chinese, *χ*^*2*^ chi-square, *df* degrees of freedom, *CFI* comparative fit index, *RMSEA* root mean square error of approximation, *Δ* changes in *χ*^*2*^, *df*, CFI, and RMSEA, *N/A* not applicable

#### Longitudinal measurement invariance

With regards to LCFA, results showed that all fit indices fitted the range, and all models were acceptable (CFI and TLI ≥ 0.900, RMSEA ≤ 0.100, SRMR ≤ 0.080; ΔCFI and ΔTLI ≤ 0.010, ΔRMSEA ≤ 0.015, ΔSRMR ≤ 0.030) (Table 4).

**Table 4:**
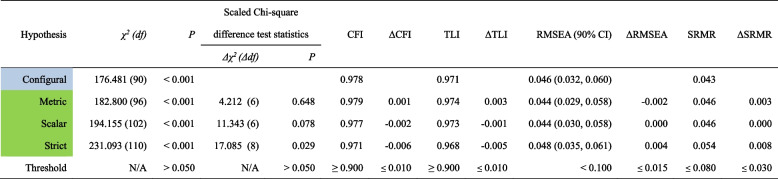
Longitudinal measurement invariance of the SCI-SC across time (*N* = 343)

Notes: Table shadings of the first column represent different levels of model held: 1) Blue represents this is the configural model; 2) Green represents that this model is fully supported

Abbreviations: *SCI* Sleep Condition Indicator, *SC* simplified Chinese, *χ*^*2*^ chi-square, *df* degrees of freedom, *CFI* comparative fit index, *TLI* Tucker–Lewis index, *RMSEA* root mean square error of approximation, *CI* confidence interval, *SRMR* standardized root mean square residual, *Δ* changes in *χ*^*2*^, *df*, CFI, TLI, RMSEA, SRMR, *N/A* not applicable

### Convergent and discriminant validity

**Fig. 1:**
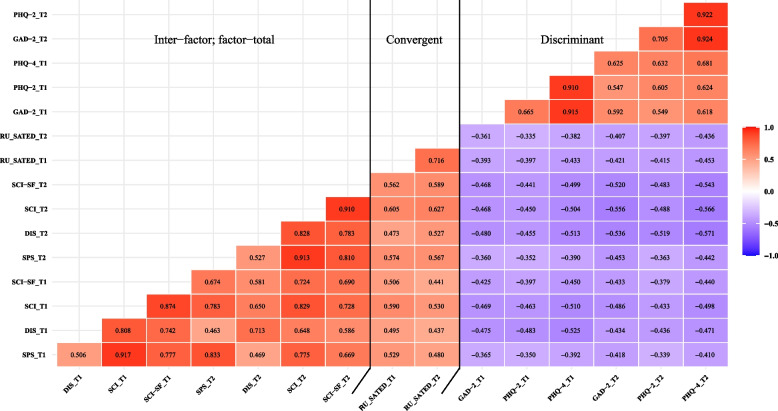
Inter–factor and factor–total, convergent and discriminant validity between the SCI-SC, RU_SATED-C scale and PHQ-4-C (*N* = 343) Notes: The color gradient indicates the correlation strength. Red indicates positive correlation and purple indicates negative correlation Abbreviations: *SCI* Sleep Condition Indicator, *SC* simplified Chinese, *SF* Short Form, *DIS* Daytime Impact Subscale, *SPS* Sleep Pattern Subscale, *RU_SATED* Regularity, Satisfaction, Alertness, Timing, Efficiency, Duration, *PHQ‑4* Patient Health Questionnaire‑4, *GAD‑2* Generalized Anxiety Disorder‑2, *PHQ‑2* Patient Health Questionnaire‑2, *T1* Time 1, *T2* Time 2

Results of the correlation analysis are reported in Fig. 1. The left side of the black line represents inter–factor and factor–total correlations, whereas the right side of the black line represents convergent and discriminant validity. The inter–factor and factor–total correlations ranged from 0.463 to 0.917, representing moderate to high correlations.

(i) A strong correlation was shown between scores on the SCI-SC and RU_SATED-C scale (*|r|*≥ 0.500) that fell within the hypothesized range, supporting for satisfactory convergent validity.
(ii) A moderate correlation was shown between scores on the SCI-SC and PHQ-4-C (0.300 ≤ |*r*| < 0.500) that fell within the hypothesized range, supporting for satisfactory discriminant validity.

### Internal consistency and test–retest reliability

Table 5 shows internal consistency results of the SCI-SC and its subscales. For two time points, both values indicated excellent internal consistency (Cronbach’s alpha and McDonald’s omega > 0.700). Supplementary 1: Table S3 displays the reliability results of other two measures.

**Table 5:** Internal consistency and test–retest reliability of the SCI-SC (*N* = 343)

	Cronbach’s alpha		McDonald’s omega		ICC (95% CI)	SEM	
	Baseline	Follow-up	Baseline	Follow-up		Baseline	Follow-up
SCI	0.811 (0.781, 0.841)	0.835 (0.809, 0.862)	0.805 (0.772, 0.837)	0.832 (0.805, 0.860)	0.829 (0.792, 0.859)	2.656	2.651
SPS	0.715 (0.667, 0.763)	0.749 (0.708, 0.789)	0.749 (0.706, 0.791)	0.782 (0.745, 0.819)	0.831 (0.794, 0.861)	1.337	1.291
DIS	0.864 (0.839, 0.889)	0.887 (0.866, 0.908)	0.864 (0.839, 0.889)	0.888 (0.867, 0.908)	0.714 (0.657, 0.762)	1.178	1.218

Notes: Standard error of measurement was calculated as “SD × sqrt (1-ICC)”

Abbreviations: *SCI* Sleep Condition Indicator, *SC* simplified Chinese, *ICC* intraclass correlation coefficient, *CI* confidence interval, *SEM* standard error of measurement, *SPS* Sleep Pattern Subscale, *DIS* Daytime Impact Subscale, *SD* standard deviation

Meanwhile, ICC values spanned two-time points higher than 0.700, and SEM values ranged from 1.178 to 2.656, indicating excellent test–retest reliability (Table 5).

## Discussion

This study provided a novel perspective on the measurement properties of the SCI-SC in a sample of healthcare students that has a comparable distribution of gender and age to general Chinese healthcare students [88]. As far as we are aware, it is the first to provide LMI evidence of the SCI. The findings demonstrated an appropriate two-factor model, multi-group and longitudinal MI, reasonable convergent and discriminant validity, good internal consistency, and adequate test–retest reliability in healthcare students. In general, the SCI-SC is a reliable screening tool for appraising insomnia symptoms among healthcare students.

### Structural validity

CFA results within Chinese healthcare students demonstrated the most excellent fit indices in the two-factor model, replicating finding from the simplified Chinese version among community residents [27]. The finding is consistent with most languages: the original English [19], traditional Chinese [11], French [22], Persian [25], Indonesian [28] and Turkish [29]. However, it is worth noting that the Swedish version produced unidimensionality outcome among university students [24], attributed to the classical test theory (CTT) being sample- and culture-dependent [89]. Therefore, further assessment of factor structure of the SCI-SC in different samples is called for to further strengthen its applicability across cultures and samples.

### Measurement invariance

When testing groups with diverse backgrounds, evaluating MI is the prerequisite for meaningful comparisons across subgroups [90]. The four invariance models, configural, metric, scalar, and strict invariance, respectively, assume equality of factor structure, factor loadings, item intercepts, and item residual across groups and two time points [78]. Strict invariance was supported across gender, age, home location, physical exercise, and stress-coping strategy, suggesting that the SCI-SC was a reliable instrument to measure insomnia among different demographic characteristics in healthcare students. More specially, given confirming configural, metric, and scalar invariance, the current study findings supported reference comparisons of the measured structure, unstandardized regression coefficients, and regression coefficients and means of the latent structures of the SCI-SC in healthcare students [79]. While some researchers have questioned the impracticality of any MI level above scalar invariance [79], strict invariance is necessary to test for differences in factor structure or latent means [80]. Strict invariance was not reached between subgroups of part-time job. It is in line with some comparable literature; for instance, a study among nursing students revealed that students who have part-time jobs were at risk for sleep disorders [91]. The study determined sufficient LMI that the SCI-SC could capably measure a certain construct similarly across time. Naturally, as the study is the first to test LMI of the SCI, further studies on LCFA are desirable.

### Convergent and discriminant validity

A strong correlation was observed between the SCI-SC and RU_SATED-C scale, supporting our hypothesis that the SCI-SC had adequate convergent validity among healthcare students. The RU-SATED-C scale assesses global sleep health and contains components such as satisfaction with sleep and sleep efficiency that are closely related to insomnia, and as such, the high correlation evidence between these two scales may be attributed to their shared focus on the same aspect of sleep. Furthermore, regarding discriminant validity, a moderate correlation was found between the SCI-SC evaluating insomnia and the PHQ-4-C assessing anxiety and depression, providing sufficient evidence to support capability of the SCI-SC to differentiate between theoretically different structures [58, 92]. Additionally, the finding aligns with other surveys on the correlation of insomnia with anxiety and depression [93–96]. An international longitudinal study has revealed that post-pandemic insomnia symptoms produced the highest level of anxiety and depression [97]. As more insomnia symptoms may lead to increased mental health issues, it is important to accurately assess insomnia among healthcare students.

In the insomnia research field, the PSQI and ISI are widely used due to their good psychometric properties and easy of use [47]. However, the PSQI was designed to evaluate general sleep disturbances, not specifically for insomnia [17, 98]. The ISI was developed not based on the updated DSM-5 and lacks an assessment of duration [18, 99]. The SCI is based on the updated DSM-5 insomnia criteria, following the latest developments in the understanding of the psychopathology of insomnia and incorporating published research diagnostic criteria and recommended quantitative parameters for sleep disturbance [19]. Strong relationships between the SCI and commonly-used scales have been validated in various languages: English [19], Italian [21], traditional Chinese [11], French [22], Swedish [24], and Indonesian [28]. In contrast, there is a gap in the testing of the SCI-SC against commonly-used scales, underscoring the imperative for future research to evaluate its relationship with scales like the PSQI and ISI.

### Internal consistency and test–retest reliability

The study illustrated the strong inter–factor and factor–total correlations, excellent internal consistency, and stable test–retest reliability of the SCI-SC among healthcare students. This effectiveness as a screening tool for insomnia symptoms was consistent with a previous report among Chinese community residents [19, 27]. Due to criticism of Cronbach’s alpha from some scholars [100], this study also used McDonald’s omega to corroborate internal consistency. Our findings suggested high inter-item relatedness and have evidence to support the high degree of interrelatedness among items [83]. Our ICC and SEM analyses supported robustness of test-retest reliability.

### Strengths and limitations

Some strengths are worth mentioning. First, we did not merely validate reliability and validity of the SCI-SC but also explore detailed MI across multiple groups. Second, this study conducted repeated measurements over two time points, rare in studies of the SCI. Last, this study is valuable for application among healthcare students and an addition to LMI of the SCI.

Some limitations deserve comment. Although adequate convergent and discriminant validity have been reported by establishing the correlation of the SCI-SC with the RU_SATED-C scale and dissociation from the PHQ-4-C, there was a lack of comparison with commonly-used scales, e.g., the PSQI and ISI. Moreover, convenience sampling method in only healthcare students and a single university site may all lead to a reduction in generalizability and selection bias, narrowing the extensiveness of conclusions.

### Future directions

Results supported the two-factor solution was preferred model, with high item interrelatedness and temporal stability, as well as satisfactory convergent and discriminant validity. Meaningful comparisons can be performed across groups (gender, age, home location, physical exercise, and stress-coping strategy) and over time among healthcare students, but comparisons need to be made with caution with varying part-time job situations. The SCI-SC, as an easy-to-use instrument for early screening of insomnia, could aid in clinical settings to monitor sleep among healthcare students of heightened risks. Accurately measuring insomnia using suitable tools is essential to save resources as well as early diagnosis and treatment of insomnia in healthcare students.

Subsequent studies should be appropriately considered in three ways while assessing or applying the SCI-SC: (i) an exploration with objective measures of sleep such as polysomnography, actigraphy, and smart mattresses; (ii) a validation of the SCI-SC with a more general population; (iii) a future exploration with multi-approaches, for instance, item response theory (IRT) and network analysis.

## Conclusion

This study demonstrated promising measurement properties of the SCI-SC in healthcare students as the first assessment of LMI and test–retest reliability. Through adaptation and validation, a wealth of valid and reliable evidence was found for use of the SCI-SC in screening insomnia symptoms. The SCI-SC, with good psychometric properties, is instrumental in establishing DSM-5-based insomnia diagnosis and treatment in healthcare students.

### Supplementary Information

Supplementary Material 1.

Supplementary Material 2.

## Data Availability

The datasets analyzed during the current study are not publicly available due to the personal health information of participants needing to be protected but are available (de-identified data) from the corresponding author on reasonable request.

## Abbreviations

CFA: confirmatory factor analysis
CFI: comparative fit index
COSMIN: COnsensus-based Standards for the selection of health Measurement INstruments
DI: Daytime Impact
DIS: Daytime Impact Subscale
DSM-5: Diagnostic and Statistical Manual of Mental Disorders, Fifth Edition
GAD-2: Generalized Anxiety Disorder-2
ICC: intraclass correlation coefficient
LCFA: longitudinal confirmatory factor analysis
LMI: longitudinal measurement invariance
MI: measurement invariance
PHQ-2: Patient Health Questionnaire-2
PHQ-4: Patient Health Questionnaire-4
Q-SV: sociodemographic variables questionnaire
RU_SATED: Regularity, Satisfaction, Alertness, Timing, Efficiency, Duration
RMSEA: root means square error of approximation
SCI: Sleep Condition Indicator
SP: Sleep Pattern
SPS: Sleep Pattern Subscale
SRMR: standardized root mean square residual
TLI: Tucker–Lewis index
