# Supplementary material for: A longitudinal examination of the measurement properties and invariance of the Sleep Condition Indicator in Chinese healthcare students
Source: BMC Psychiatry. 2024 Jul 22;24:518. doi: 10.1186/s12888-024-05844-7 (PMC11264982; doi:10.1186/s12888-024-05844-7)
Supplement: Supplementary file 2 — Supplementary Material 2. [file 12888_2024_5844_MOESM2_ESM.docx]

## Measurement invariance

### *Cross-sectional measurement invariance*

Cross-sectional measurement invariance on two-factor model, the best-fitting model within three alternative SCI-SC models was tested. Table 3 and Supplemental 1: Table S2 list measurement invariance results across gender, age, home location, part-time job, physical exercise, and stress-coping strategy on Chinese healthcare students. For all models, if at least two out of three fit indices comply with the cut-off criteria, it indicates that MI is held [1]. The following results are the detailed analysis for each subgroup.

**Gender:** For the baseline sample, when parameters were freely estimated between male and female subgroups, configural invariance provided acceptable fit data (CFI and TLI ≥ 0.900, RMSEA ≤ 0.100, and SRMR ≤ 0.080). We then moved on to test metric, scalar, and strict invariance in healthcare students. Results showed that changes in fit indices were all within the cut-off criteria (ΔCFI and ΔTLI ≤ 0.010, ΔRMSEA ≤ 0.015, and ΔSRMR ≤ 0.030), maximum supporting strict invariance across gender. For the follow-up sample, the configural invariance was satisfied (CFI and TLI ≥ 0.900, RMSEA ≤ 0.100, and SRMR ≤ 0.080). Although some of specific values exceeded recommendation, due to over two-thirds of fit indices, strict invariance was supported (ΔCFI ≤ 0.010, ΔRMSEA ≤ 0.015, and Δ SRMR ≤ 0.030).

**Age:** For the baseline sample, CFI, TLI, RMSEA, and SRMR all remained in an acceptable range, supporting configural invariance across < 20 and ≥ 20 years subgroups (CFI and TLI ≥ 0.900, RMSEA ≤ 0.100, and SRMR ≤ 0.080). Maximum strict invariance was accepted due to over two-thirds of fit indices (ΔTLI ≤ 0.010, ΔRMSEA ≤ 0.015, and ΔSRMR ≤ 0.030). Although both ΔCFI and ΔTLI exceeded cut-off values, scalar invariance was still supported due to negligible changes in fit indices for strict invariance. For the follow-up sample, the resulting strict invariance showed a good fit to the data over two-thirds of fit indices (ΔTLI ≤ 0.010, ΔRMSEA ≤ 0.015, and ΔSRMR ≤ 0.030).

**Home location:** For the baseline sample, configural invariance solution across three subgroups (urban, rural, and suburban) displayed an excellent fit (CFI and TLI ≥ 0.900, RMSEA ≤ 0.100, and SRMR ≤ 0.080), also in metric, scalar, and strict invariance (ΔCFI and ΔTLI ≤ 0.010, ΔRMSEA ≤ 0.015, and ΔSRMR ≤ 0.030). For the follow-up sample, similar results were obtained, the highest supporting strict invariance over two-thirds of fit indices (ΔCFI ≤ 0.010, ΔRMSEA ≤ 0.015, and ΔSRMR ≤ 0.030).

**Part-time job:** For the baseline sample, configural invariance was satisfied with acceptable fit indices (CFI and TLI ≥ 0.900, RMSEA ≤ 0.100, and SRMR ≤ 0.080). Furthermore, scalar invariance across part-time job (yes and no) subgroups were supported (ΔCFI and ΔTLI ≤ 0.010, ΔRMSEA ≤ 0.015, and ΔSRMR ≤ 0.030), but no strict invariance due to large changes in CFI and TLI values. For the follow-up sample, up to scalar invariance was accepted (ΔCFI and ΔTLI ≤ 0.010, ΔRMSEA ≤ 0.015, and ΔSRMR ≤ 0.030).

**Physical exercise:** For the baseline sample, configural invariance across physical exercise (yes and no) subgroups displayed a good fit to the data (CFI and TLI ≥ 0.900, RMSEA ≤ 0.100, and SRMR ≤ 0.080). No decreases in fit indices exceeded the recommended cutoff values when adding restrictions to the model, scalar and strict invariance were fully supported, and metric invariance could be accepted as over two-thirds of fit indices (ΔCFI and ΔTLI ≤ 0.010, ΔRMSEA ≤ 0.015, and ΔSRMR ≤ 0.030). For the follow-up sample, all models displayed acceptable changes in fit indices to the data (CFI and TLI ≥ 0.900, RMSEA ≤ 0.100, and SRMR ≤ 0.080; ΔCFI and ΔTLI ≤ 0.010, ΔRMSEA ≤ 0.015, and ΔSRMR ≤ 0.030).

**Stress-coping strategy:** For the baseline sample, configural invariance across stress-coping strategy subgroups (emotion-focused, solution-focused and avoidance coping) were accepted by fit indices meeting the requirement for acceptable fit (CFI and TLI ≥ 0.900, RMSEA ≤ 0.100, and SRMR ≤ 0.080). The assumptions of all invariance were satisfied with fit indices within the recommended range due to over two-thirds of fit indices (ΔCFI and ΔTLI ≤ 0.010, ΔRMSEA ≤ 0.015, and ΔSRMR ≤ 0.030). For the follow-up sample, configural, metric, scalar, and strict invariance were supported by the acceptable fit indices showing according to over two-thirds of fit indices (CFI and TLI ≥ 0.900, RMSEA ≤ 0.100, and ΔSRMR ≤ 0.030; ΔCFI and ΔTLI ≤ 0.010, ΔRMSEA ≤ 0.015, and ΔSRMR ≤ 0.030).

In light of the secondary indicator of *χ^2^* value and *P* value were also used as reference standards, Δ*χ^2^* results indicated no significant difference (*P* > 0.050) between most subgroups.

# Reference

1. Nelemans SA, Meeus WHJ, Branje SJT, Van Leeuwen K, Colpin H, Verschueren K, Goossens L. **Social Anxiety Scale for Adolescents (SAS-A) short form: longitudinal measurement invariance in two community samples of youth**. *Assessment.* 2019; **26**(2):235-48. doi:10.1177/1073191116685808
